# A Study on the Development of the Stainless Steel Tube Bundle Structure Detecting System Using Ultrasonic Guided Wave

**DOI:** 10.3390/s24165278

**Published:** 2024-08-15

**Authors:** Jeongnam Kim, Jiannan Zhang, Azamatjon Kakhramon ugli Malikov, Younho Cho

**Affiliations:** 1 Department of Reliability, Virtual Engineering Platform Research Division, Korea Institute of Machinery & Materials, 156 Gajeongbuk-ro, Yuseong-gu, Daejeon 34103, Republic of Korea; kjnnoah@kimm.re.kr; 2 Graduate School of Mechanical, Pusan National University, 63 Beon-gil, Busandaehak-ro, Geumjeong-gu, Busan 46241, Republic of Korea; zjn1997@pusan.ac.kr (J.Z.); malikov@pusan.ac.kr (A.K.u.M.); 3 School of Mechanical Engineering, Pusan National University, 63 Beon-gil, Busandaehak-ro, Geumjeong-gu, Busan 46241, Republic of Korea

**Keywords:** tube bundle, nondestructive testing, guided wave, fast transform, narrow gap

## Abstract

In this study, an ultrasonic guided wave system that can be used to detect broken tubes in stainless steel tube bundle structures (e.g., heat exchangers) with fairly narrow spacing between the tubes was designed. The interval between the tubes was 1.5 mm, and the thickness of the strip with a transducer that can be inspected by passing between the tubes was designed to be 1 mm. The damaged specimen was filled with water, and it was confirmed that the signal amplitude was smaller than that of the normal specimen filled with air. The ultrasonic properties of stainless steel were analyzed using the developed system, and it is expected that this will contribute to breakage inspection for tube bundles with narrow spacing.

## 1. Introduction

A tube bundle is a collection of small-diameter tubes held together by a tube sheet or support plate, commonly used in industrial applications to transfer heat or coolants between fluids. This group of pipes is usually housed in a tubular shell or outer housing, through which water flows constantly. When a hot liquid or gas is circulated through the tube bundle, the large mating surface area of the tube can efficiently transfer heat to the shell water and cool the fluid in the tube. In power plants, shipbuilding, offshore, petrochemical plants, and nuclear power plants, tube bundle structures are mainly used for heat exchange or the transportation of gas and oil. Pipes exposed to these aquatic environments are particularly difficult to manage due to threats such as external shock, corrosion, and aging, and if they leak or break, they can cause enormous economic loss and environmental risks. Furthermore, repairing pipes exposed to the aquatic environment is difficult, costly, and time-consuming, and is a task that requires real-time monitoring.

Nondestructive testing (NDT) is a range of techniques used to evaluate physical properties and detect defects in materials and structures without causing any damage. It involves various methods such as radiography, ultrasonic testing, electromagnetic testing, and thermal testing, which can further aid in quality inspection and safety evaluation. Currently, four types of nondestructive testing techniques are mainly applied to the inspection method of tube bundles. Among them, in the case of eddy current inspection or ultrasonic IRIS (Internal Rotary Inspection System), various sensors inserted into the tube are used to inspect local corrosion and erosion or measure the thickness. In the case of these methods, since they perform flaw detection on local points, they require a lot of time and money, and it is difficult to detect defects in the tube because the inspection is performed only on areas accessible to humans or machines.

Therefore, as an alternative to conventional ultrasonic testing, ultrasonic guided wave, which can perform a precise diagnosis according to the shape of a complex structure, can be utilized. Ultrasonic guided wave has the advantage of being able to detect a wide range within a short time compared to bulk waves, so it is mainly used for inspection of tubes and plates. In addition, in the case of bulk waves, inspection is performed only on areas that can be accessed by humans or machines, whereas in the case of an ultrasonic guided wave, it is possible to inspect inaccessible areas or complex structures.

Rose conducted a lot of research on long-distance piping inspection using ultrasonic guided waves [1,2,3,4,5]. Some of the research is on the development of transducers or sensors for inspecting pipelines. Hay and Rose, while using a PVDF comb-type transducer, searched for a method to generate ultrasonic guided waves in a tube [6]. Bray, A.V. et al. developed a unique capability in the application of guided wave (GW) technology for nondestructive evaluation (NDE) of corrosion under an insulation pipe and aimed to develop methods and systems that enable on-site inspection [7]. D.H. Lee et al. proposed an optimal method to effectively detect corrosion under insulation using ultrasonic guided wave for insulated tubes through a theoretical analysis of tubes [8]. J.P. Park. et al. studied the factors affecting the propagation of ultrasonic guided waves in a curved part welded to a cylindrical pipe structure [9]. M.S. Park et al. developed an externally installed transducer that can effectively identify the location of internal defects for large-diameter water tube inspection and designed it to generate and measure only the shear wave mode [10]. Maciej W. evaluated the burst pressure and structural integrity of steel pipes using the axial excitation magnetic flux leakage technique with respect to the grouping criteria of closely spaced volumetric surface features [11].

K.C. Hwang et al. presented the development of an ultrasonic inspection system that uses guided waves to detect broken tubes in stainless steel tube bundles. The system is based on a combination of wave propagation and signal processing techniques. The authors reported on the performance of the system and demonstrated its potential for detecting broken tubes [12]. T. Watanabe et al. made progress on the development of an ultrasonic guided wave inspection system for detecting corrosion/erosion damage in tubes in nuclear power plants. The system uses guided waves to detect damage and the authors reported on its performance. They also presented a case study of the system’s application in a nuclear power plant [13]. L. Li et al. discussed the use of guided wave testing for the inspection of tubes in heat exchangers. The authors presented a method for detecting defects in tubes and reported on its performance. They also discussed the potential benefits of guided wave testing for the inspection of heat exchanger tubes [14]. Y. Su et al. studied the development and application of ultrasonic guided wave testing to detect defects in tubes. The authors presented a method to detect defects using guided waves and reported on its performance. They also discussed the potential benefits of guided wave testing for the inspection of tubes [15]. K. Song and H. Kim presented the development of a guided wave inspection system using piezoelectric transducers for detecting broken tubes in heat exchangers. The authors described the design and implementation of the system and report on its performance. They also discussed the potential benefits of the system for the inspection of heat exchanger tubes [16].

As mentioned above, there are many previous studies on the tube using guided waves [17,18,19,20,21,22,23]. Research on pipes exposed to the aquatic environment is also in progress [24,25,26,27,28,29,30]. In this case, instead of using a general transducer, the inspection was performed using a submerged ultrasonic transducer. But in most cases, the size of the transducer is relatively large. The contact-based NDT method has the disadvantage that accessibility to a specific area may be limited because the sensor or transducer must be in contact with the surface of the structure. Due to this, the inspection space is narrow because there are many tubes, and when inspecting complex structures, accessibility is not possible, so inspection is not possible. 

Therefore, the purpose of this study is to develop an inspection system using a relatively thin transducer for a structure with a narrow space, such as a heat exchanger, where the application of other nondestructive inspection techniques is limited. The proposed system not only enables inspection in a narrow space compared to other NDT techniques, but also presents a method for inspecting a wide range in a short time.

## 2. Transducer and Specimen Fabrication

**Figure 1:**
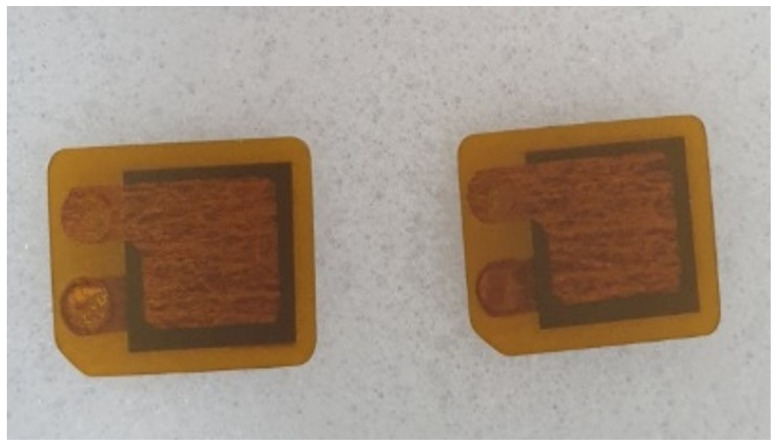
Patch-type transducer.

**Figure 2:**
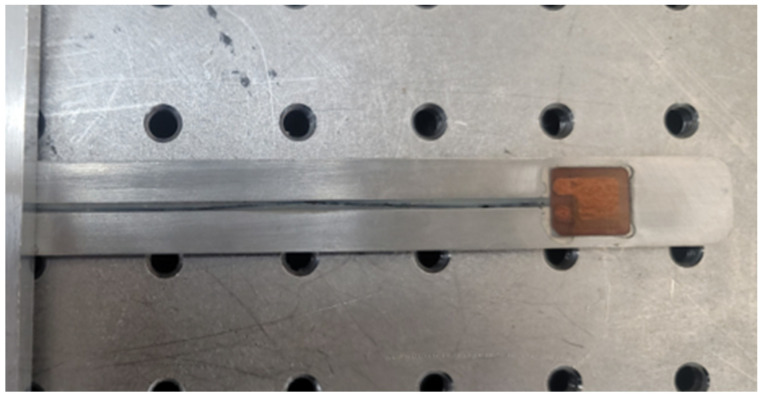
Fabricated strip transducer.

The patch-type piezoelectric transducer shown in Figure 1 is a type of piezoelectric transducer that converts electrical energy into mechanical energy and vice versa. Its structure consists of a piezoelectric ceramic material sandwiched between two metal plates. When an electrical voltage is applied to the ceramic material, it undergoes deformation, generating mechanical vibrations that produce ultrasonic waves in the surrounding medium. Conversely, when ultrasonic waves propagate through the medium and reach the transducer, they induce vibrations in the ceramic material, which generate an electrical signal that can be measured by an external instrument. The working principle of the transducer is based on the piezoelectric effect, which is the ability of certain materials to generate an electrical charge in response to mechanical stress or deformation. In the case of this transducer, it is made with piezoelectric lead zirconate titanate (PZT), a ceramic material with high piezoelectric coefficients that make it suitable for generating and detecting high-frequency ultrasonic waves. And it was manufactured by attaching it to a stainless steel strip, as shown in Figure 2. Epoxy was used to fix the cable welding area so that it did not move with the waterproofing. The final thickness of the designed strip transducer is 1 mm. 

**Figure 3:**
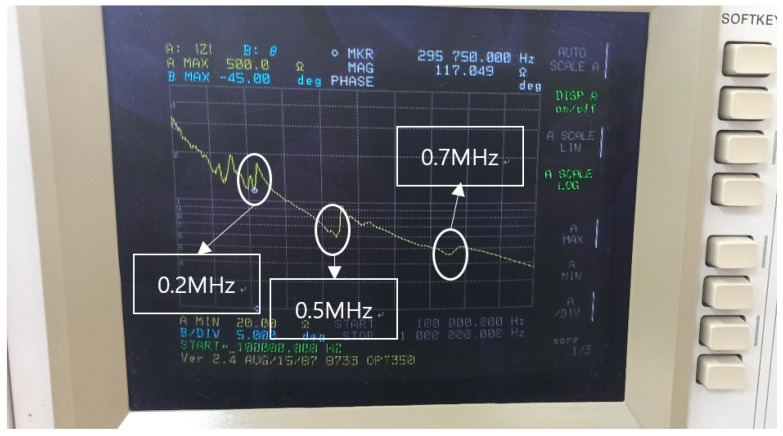
Resonance frequency.

The characteristics of transducers are distinguished by the presence of electrical resonant and anti-resonant frequencies. Theoretically, the resonant frequency is the operating frequency at which a piezoelectric transducer attains the minimum impedance and converts electrical energy to mechanical energy most efficiently. The easiest way to measure the characteristics of piezoelectric transducer components is with an impedance analyzer. This is the result of measuring the impedance of the piezoelectric material using the impedance analyzer HP 4194 A in Figure 3. The resonant frequencies of this transducer were investigated and found to be 0.2 MHz, 0.5 MHz, and 0.7 MHz. In this study, a frequency of 0.2 MHz, which has a relatively high energy level, was used for the experiment. Since it was difficult to predict which resonant frequency would respond most effectively to the defect during the experimental design, we used a frequency of 1 MHz to simultaneously observe the response at three resonant frequencies: 0.2 MHz, 0.5 MHz, and 0.7 MHz.

**Figure 4:**
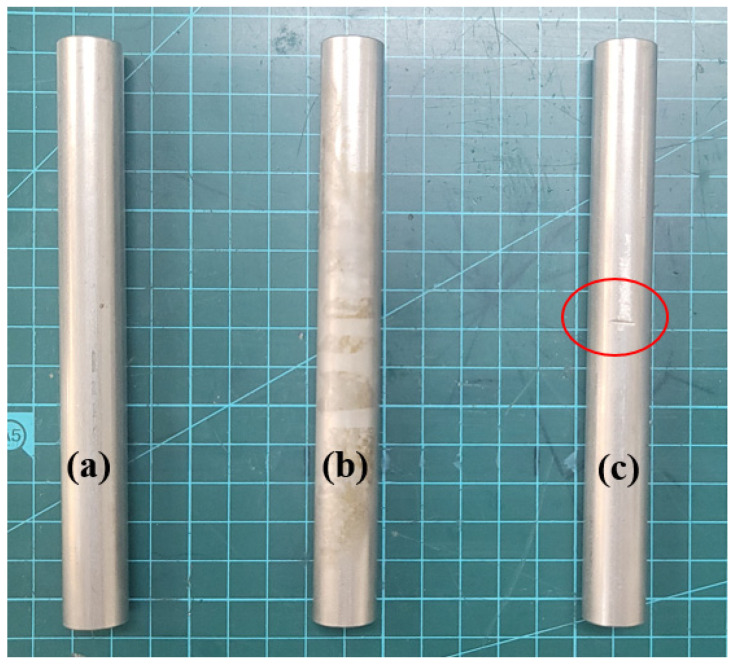
Stainless steel tube specimens. (**a**) Normal, (**b**) corroded, and (**c**) damaged. Specimen (**c**) was made with artificial defects 0.3 mm deep (red circle); these were made with a grinder.

As shown in Figure 4, three types of specimens were prepared. All specimens were made of stainless steel. The defective specimen (b) was exposed to an acidic solution containing less than 9% hydrochloric acid for 3 h to corrode the surface. Specimen (c) was made with artificial defects 0.3 mm deep; these were made with a grinder. Defective specimens were prepared so that defects corresponding to the propagation path of ultrasonic waves could be located.

## 3. Ultrasonic Guided Wave in Tube-like Structure

Ultrasonic guided wave is a technology that can find defects up to several tens of meters away from the point where the signal is generated. The velocity of ultrasonic waves varies according to the properties of the material, and ultrasonic guided waves have dispersion properties in which the propagation speed varies according to the frequency. Therefore, it is important to calculate and confirm the dispersion curve for the test object before applying it to the tube.

The equation of motion in an isotropic homogeneous elastic tube is expressed by Equation (1), called the Navier equation of motion.

(1) $$\mu \nabla^{2}\vec{u}+\left(\lambda +\mu \right)\nabla \left(\nabla \cdot \vec{u}\right)=\rho \frac{\partial^{2}\vec{u}}{\partial t^{2}}$$

where λ and μ are Lame constants, ∇^2^ is the 3D Laplace operator, $\vec{u}$ is the displacement vector, and ρ is the density.

**Figure 5:**
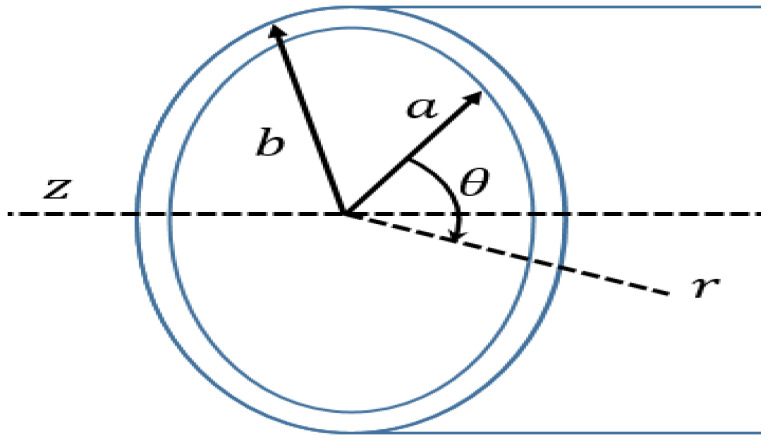
Tube coordinates used for segmentation diagram analysis.

As shown in Figure 5, due to the boundary conditions on the inner and outer surfaces of the tube, when r = a and r = b, the traction is the same as Equation (2).

(2) $$\sigma_{rr}=\sigma_{r\theta }=\sigma_{rz}=0,\;(r=a,\;b)$$

If Equation (1) is solved for displacement u using the Helmholtz decomposition theorem, it can be expressed as a vector and a scalar component as in Equation (3).

(3) $$\vec{u}=\nabla \Phi +\nabla \times \vec{H}$$

where **Φ** is the scalar potential, and $\vec{H}$ represents the vector potential function.

By substituting Equation (3) into Equation (1), it can be summarized as a wave equation like Equations (4) and (5).

(4) $$\nabla^{2}\Phi =\frac{1}{C_{L}^{2}}\frac{\partial^{2}\Phi }{\partial t^{2}}$$

(5) $$\nabla^{2}\vec{H}=\frac{1}{C_{T}^{2}}\frac{\partial^{2}\vec{H}}{\partial t^{2}}$$

where *C_L_* is the longitudinal wave velocity and *C_T_* is the transverse wave velocity. A solution satisfying the equation of motion in an isotropic homogeneous elastic tube was derived in detail by Gazis [31] and using Equation (2), when r = a and r = b, the frequency equation can be obtained as in Equation (6).

(6) $$D=\left|C_{ij}\right|$$

where $C_{ij}$ is a 6 × 6 linear modulus matrix. Based on this, in order to have a nontrivial solution, an eigen solution can be obtained using the condition *D* = 0. Using this, a dispersion diagram can be obtained.

**Figure 6:**
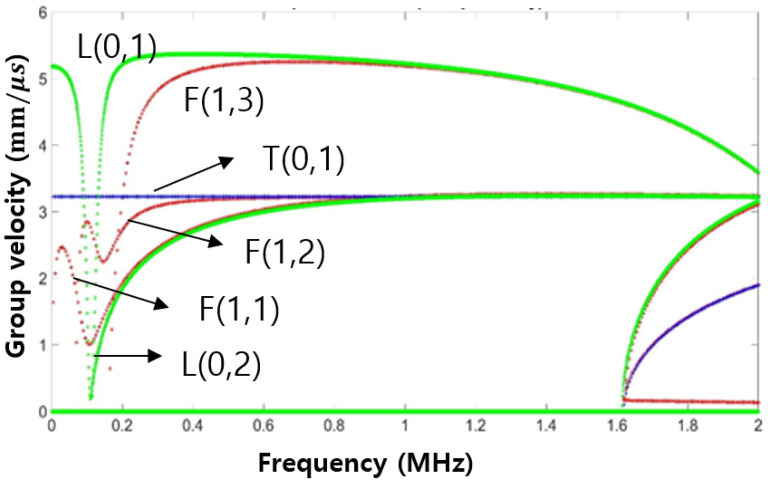
Dispersion curve group velocity for tube specimen.

In this study, an experiment was conducted using tubes made of stainless steel with an outer diameter of 16 mm, thickness of 1 mm, and length of 130 mm. Also, the dispersion curve for these tubes is shown in Figure 6 in dispersion diagrams for group velocity, respectively, and was calculated for mode analysis of the signal. Looking back at the modes shown in Figure 3, it can be seen that L(0,2) and F(1,1) modes can be generated together when the stainless tube to be inspected is excited with the corresponding transducer.

## 4. Experiment and Results

### 4.1. Experiment Setup 

**Figure 7:**
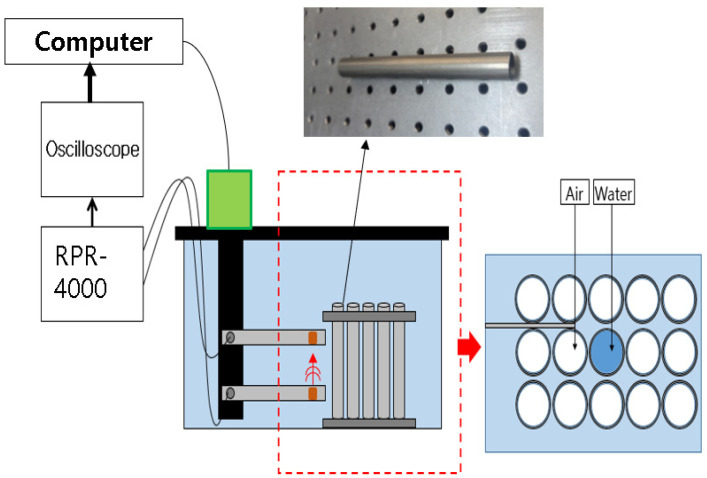
Tube bundle inspection system schematic diagram.

Figure 7 is a schematic diagram of the experiment. A signal of 1 MHz was generated using the RPR-4000 tone-burster. The signals collected by the oscilloscope were transmitted to a computer for signal processing. In the process of moving the sensor of the ultrasonic guided wave system, a computer was used instead of a person to control the movement to an accurate distance. To this end, a motor and a computer were connected to enable precise adjustment of the sensor position, and the distance between the specimen and the sensor was precisely measured and recorded in a database. The sensor was moved to an accurate position based on the distance data measured through the computer control system. The position of the sensor was finely adjusted by fine adjustment of the motor so that it could also approach the tube in the center of the bundle. A total of 15 tubes were fabricated in a 3 × 5 arrangement with 1.5 mm spacing between the tubes arranged in a tube bundle. When a pipe was damaged in an underwater environment, considering the situation in which external water flows into the pipe, it was assumed that 1 of the 15 pipes was damaged, and the experiment was conducted by filling the pipe with water.

In this measurement method, the two patch-type transducers are used, with one acting as the transmitter and the other as the receiver. The transmitter excites the ultrasonic signal, which is propagated through the water to the tube to be inspected. The ultrasonic signal then propagates along the length of the tube and reaches the receiver, which collects the signal.

**Figure 8:**
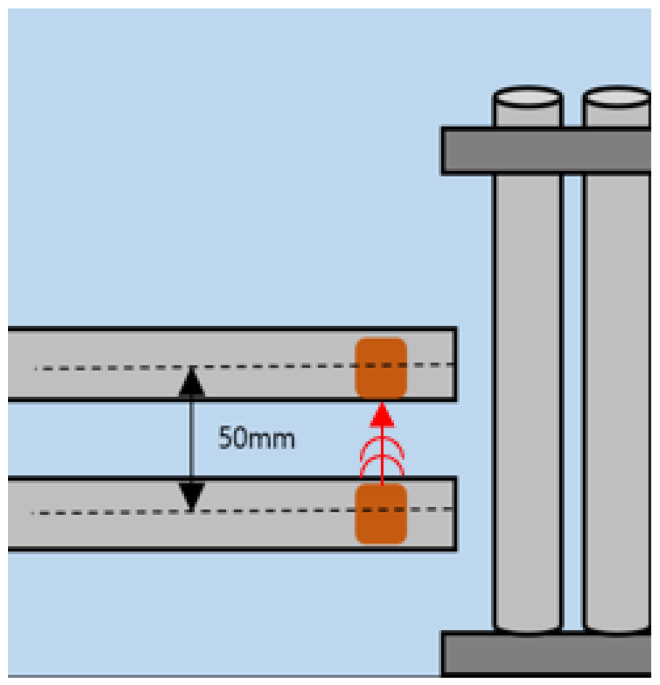
Distance between strip transducers—50 mm.

**Figure 9:**
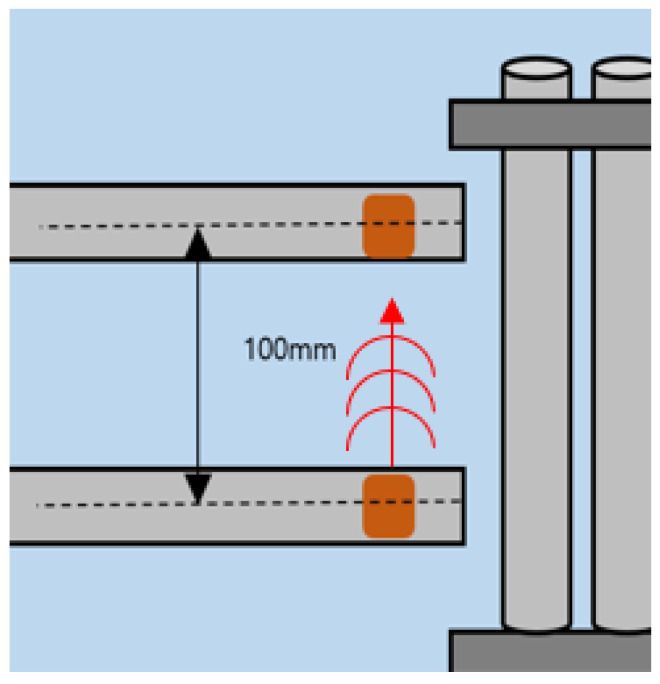
Distance between strip transducers—100 mm.

As shown in Figure 8 and Figure 9 the distance between the transmitter and receiver is 50 mm and 100 mm, respectively. And they are arranged on the same plane in the longitudinal direction of the tube to be inspected. The distance between the transducers and the tube is about 1 mm. The ultrasonic signal generated by the transmitter is propagated to the tube through the water, and the signal that is reflected or scattered by the tube is received by the receiver.

By analyzing the collected signals, it is possible to detect defects or anomalies in the tube, such as corrosion or cracks. The time of flight and amplitude of the received signal are used to determine the location and size of the defect. The overall principle of the measurement method is based on the fact that ultrasonic waves can be used to detect changes in the material properties of the tube, which can indicate the presence of defects or anomalies.

To conduct experiments to detect various defects, the distance between the two transducers was set to 50 mm. Assuming that water had leaked due to damage, an experiment was conducted by filling one specimen with water. All types of specimens, including those with corroded surfaces and those with artificial defects, were conducted using the same experimental method. And FFT (fast Fourier transform) was performed to analyze the difference between the two signal magnitudes and amplitude values.

### 4.2. Experiment Results

**Figure 10:**
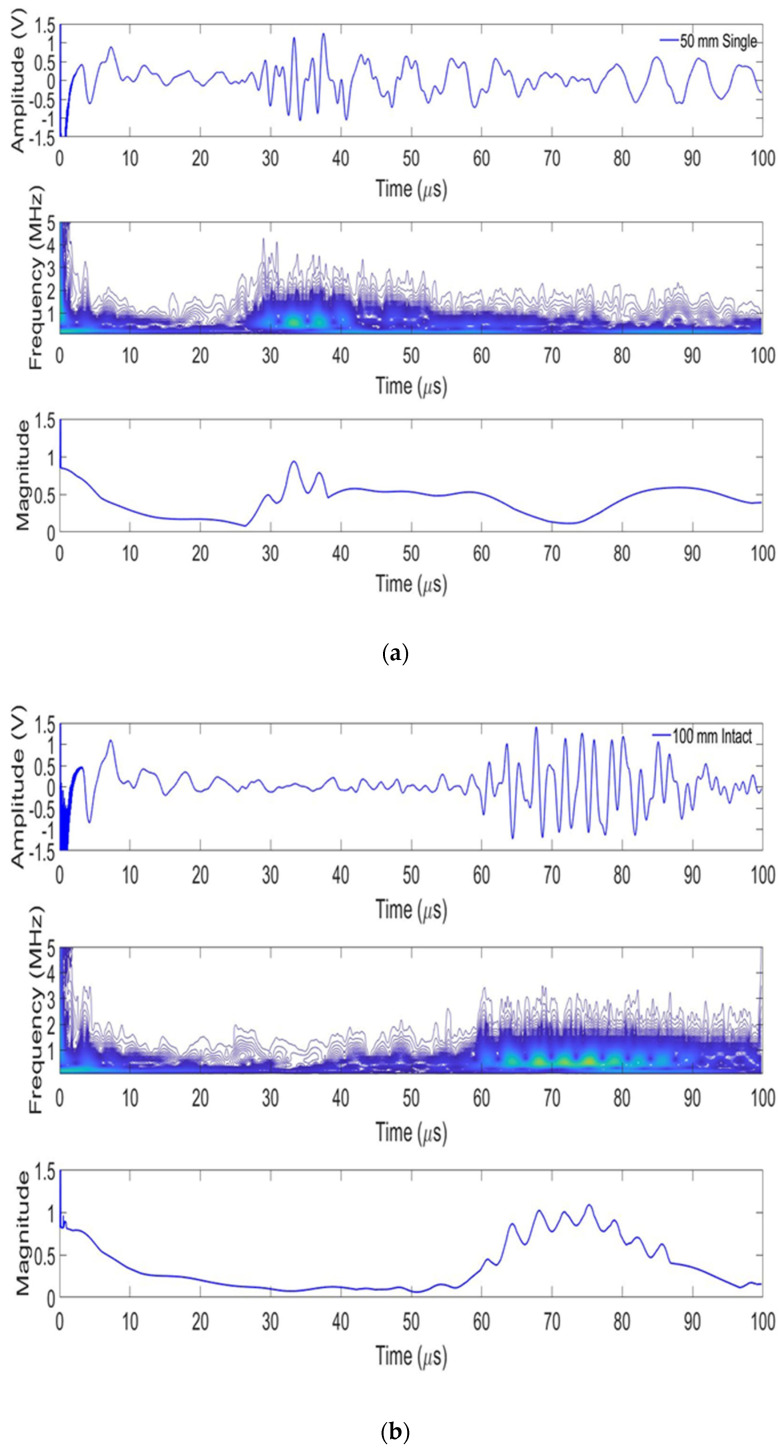
Signal for single normal tube at distance of (**a**) 50 mm and (**b**) 100 mm.

For a typical single tube, the ultrasonic signal is shown in Figure 10 when the spacing of the transmitting/receiving transducers is 50 mm and 100 mm. Based on the starting point (green lines) of the two signals, the time difference was about 30 μs, and the speed of the ultrasonic was calculated to be about 1.67 mm/μs.

**Figure 11:**
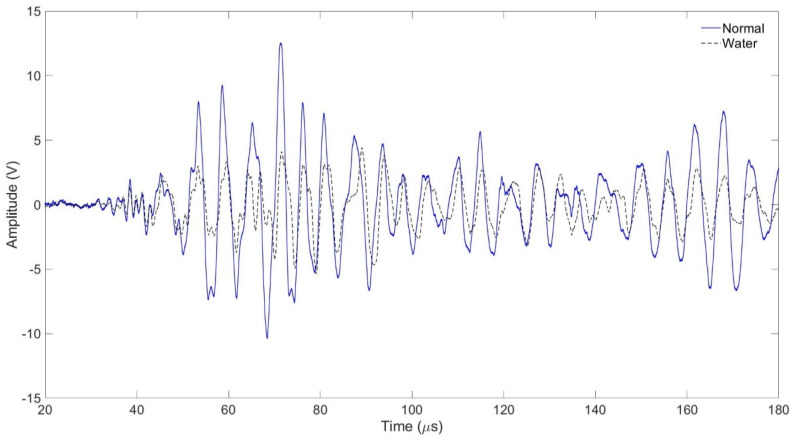
Normal tube and water-containing tube with signal received at 50 mm.

**Figure 12:**
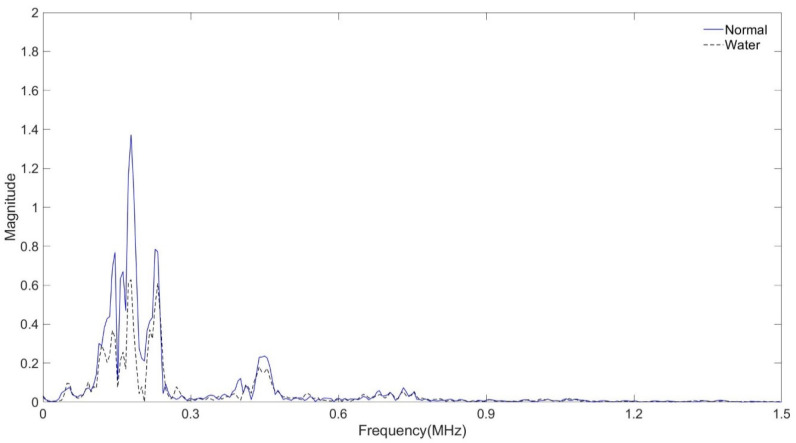
Normal tube and water-containing tube with FFT magnitude.

In the tube assembly, the experiment was carried out with the interval between the transmitting and receiving transducers being 50 mm. In the case of a normal tube in the same position, it is filled with air, and in the case of a tube that is assumed to be damaged, it is filled with water, and the signal is shown in Figure 11 and Figure 12 As shown in Figure 10, the amplitude value of the normal tube filled with air was larger than the amplitude value of the tube filled with water. Figure 11 shows the result of the FFT magnitude of the signal. In both cases, frequencies of 0.2 MHz and 0.5 MHz appeared, and at 0.2 MHz, the amplitude value of a normal tube was found to be twice as high as the damaged one, which could also be seen in Figure 11. However, the amplitude values at 0.5 MHz were similar in both cases.

**Figure 13:**
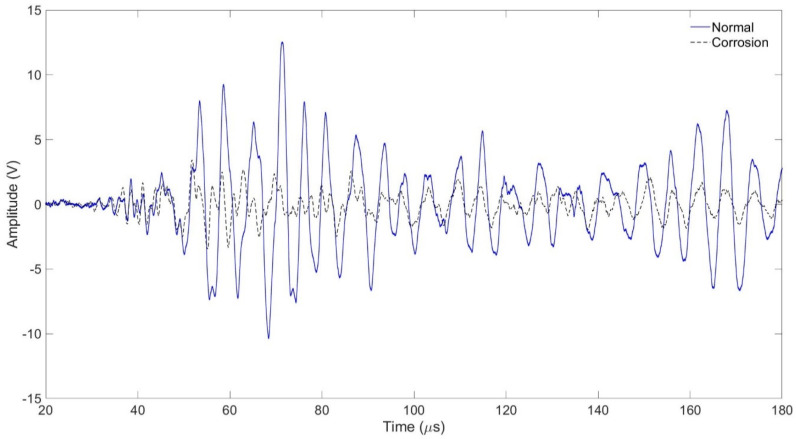
Normal tube and corrosion tube with signal received at 50 mm.

**Figure 14:**
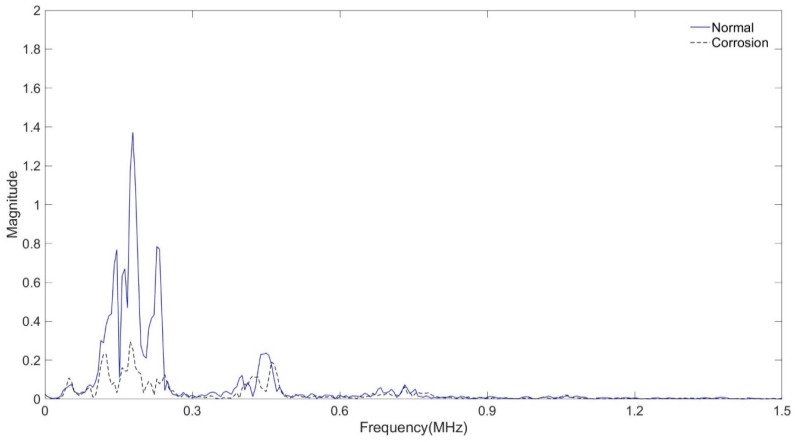
Normal tube and corrosion tube with FFT magnitude.

Signals from normal and corroded tubes are compared in Figure 12 and Figure 13. In Figure 13 the amplitude of the corroded specimen is greatly reduced, and it shows a larger difference compared to the water-filled specimen in Figure 11. In Figure 14 the amplitude of the corroded specimen shows a difference of about six times compared to the amplitude value of the general specimen.

**Figure 15:**
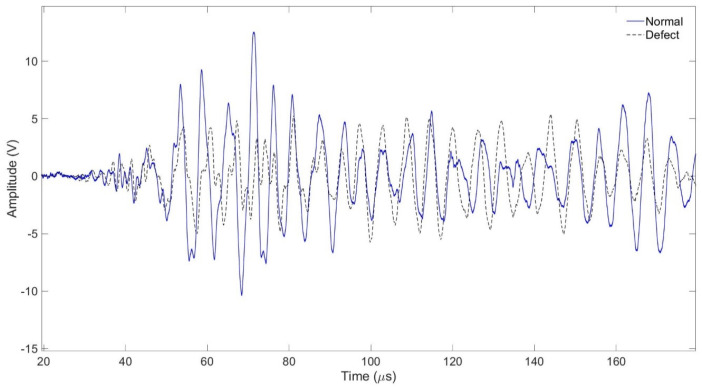
Normal tube and damaged tube with signal received at 50 mm.

**Figure 16:**
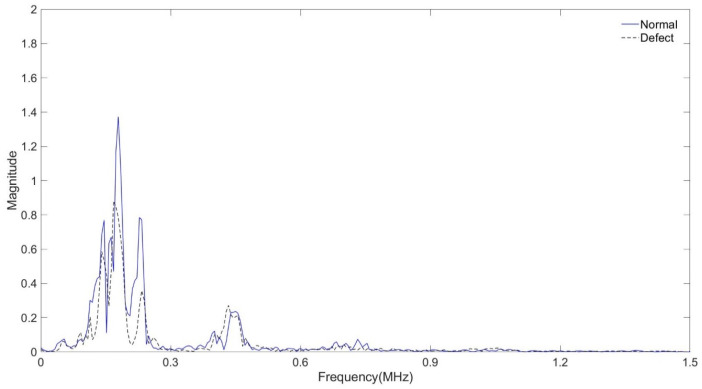
Normal tube and damaged tube with FFT magnitude.

Figure 13 and Figure 15 show a comparison of signals collected from a normal tube and a damaged specimen. In Figure 15 the difference between the two signals is smaller than in Figure 11 and in Figure 15 it is confirmed that the amplitude difference is smaller in Figure 12 It can be confirmed that the trend is similar to the results of the previous two experiments before Figure 16.

**Figure 17:**
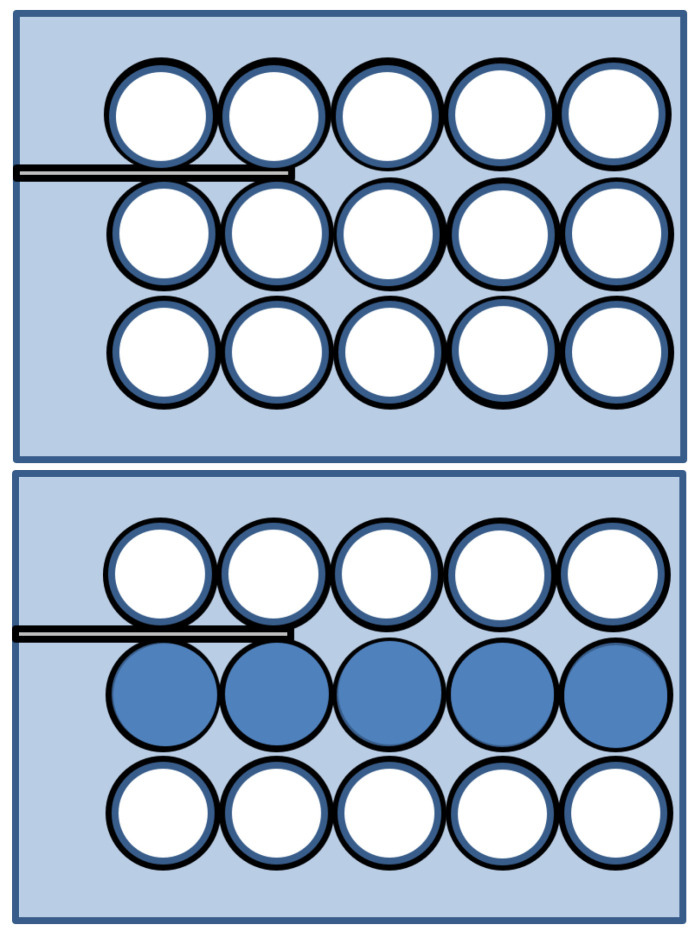
Additional experiment for normal tubes and tubes filled with water at the 2nd line.

Based on the above method, regular tubes and water-filled tubes were additionally tested for the middle row, which is difficult to test, as shown in Figure 17. The experimental results are shown in Table 1. Based on the value of the largest defective specimen among the amplitude ratios with the general tube, those that are less than 70% full are considered defective.

## 5. Discussions

In this study, a method for conducting ultrasonic guided wave testing of complex and narrow structures was presented. As a result of the experiment, the speed of the ultrasonic guided wave propagating in the longitudinal direction of the tube was confirmed to be 1.67 mm/μs. As a result of FFT magnitude, a frequency component of 0.2 MHz was also confirmed. Modes corresponding to group velocity and frequency values were observed in both L(0,2) mode and F(1,1) mode on the dispersion diagram. Based on this result, it can be inferred that the currently generated ultrasonic guided wave is not a single mode, but has two overlapping modes.

The amplitude of the signal of the water-filled specimen was smaller than that of the air-filled specimen. It was confirmed that this decreased due to the damping effect of water. In the case of the specimen with corrosion and defects, the amplitude value was smaller than that of the general tube. As a result of FFT magnitude, the amplitude value of 0.5 MHz was similar, but it was confirmed that there was a large difference in the amplitude value of 0.2 MHz. It was confirmed that it was possible to determine whether the tube was damaged or not using the signal and FFT magnitude because they sensitively respond to changes in the internal/external environment of the pipe or the type of surface defects.

## 6. Conclusions

Ultrasonic guided wave inspection technology is an important technology in the field of nondestructive inspection, which can detect defects from long distances, shorten inspection time in complex structures, and quickly inspect wide areas. It has been proven that the method of determining the presence of defects through FFT analysis of the signal is useful. This study has expanded the applicability of ultrasonic guided wave technology and presented a practical methodology for defect detection in narrow-space structures. This increases practicality in industrial sites and makes an important contribution that can complement existing nondestructive inspection technologies. 

However, ultrasonic guided wave systems have limitations in that the complexity of signal interpretation increases due to the attenuation of high-frequency signals and interference between wave modes. These problems can make precise defect detection difficult. In future studies, it is expected that improved results can be obtained by conducting intensive experiments using frequencies corresponding to frequency components that have changes through the results of frequency analysis using FFT in the system.

## Figures and Tables

**Table 1:** Test results of water-filled specimen test.

	No. 1	No. 2	No. 3	No. 4	No. 5
Magnitude of normal tube at 0.2 MHz	0.007131	0.008468	0.007604	0.001378	0.01448
Magnitude of tube filled with water at 0.2 MHz	0.004572	0.003757	0.005047	0.007313	0.008324
Amplitude ratio	64.1%	44.4%	66.7%	53.1%	57.5%

## Data Availability

The original contributions presented in the study are included in the article, further inquiries can be directed to the corresponding authors.
